# Supplementary material for: The effects of mating and blood feeding on the immune defense of female Aedes aegypti mosquitoes
Source: PLoS Negl Trop Dis. 2025 Oct 3;19(10):e0013542. doi: 10.1371/journal.pntd.0013542 (PMC12507272; doi:10.1371/journal.pntd.0013542)
Supplement: S1 Protocol — (DOCX) [file pntd.0013542.s001.docx]

**S1 Protocol**

Mosquito rearing, adult collection, and mating procedures

THAI strain females were reared under the same conditions as described for LVP in the main manuscript methods. However, populations were thinned to 200-250 first instar larvae. Adult collection was conducted using identical procedures used for LVP strain females. While all other steps in the mating procedure were also identical to those used for LVP individuals, THAI females were allowed to mate with males for 50-52 hours.

Infection with *S. marcescens*

When infecting THAI strain mosquitoes, *S. marcescens*-GFP was grown as described in the main manuscript methods, but was washed thrice by pelleting via centrifugation at 5,000 RPM for 3 minutes and resuspended in sterile 1X PBS to a final optical density of 1.0 $\pm$ 0.05 O.D._600_. This culture was then diluted with sterile 1X PBS by 1:600. Females were cold anesthetized and injected with 69.0nL (47.18±2.93 CFU) of the diluted *S. marcescens*-GFP liquid culture using a Nanoject II manual injector (Drummond scientific) fitted with a pulled Nanoject II glass capillary (Drummond scientific). A subset of 5 females per experimental group, per replicate were injected with sterile 1X PBS to ensure that mosquito death was not a result of wounding alone. This experiment resulted in 100% survival, and these data can be found in supplementary file S5. Both sterile and bacteria injected females were monitored for survival up to 24 hours post-injection.

Survival and bacterial load monitoring

Bacterial load measurements were conducted using the same procedure as described for LVP females.

For one replicate of bacterial load of THAI females, we did not plate undiluted homogenate, though diluted plates exhibited no growth. Therefore, we are unable to determine whether three zeros in this dataset (2/8 virgin values, and 1/9 mated values) are true zeros. However, inclusion of these points made no difference in the qualitative results of our analysis. Therefore, we opted to keep these three points in our dataset.

Identical to our Low dose LVP experiment, we collected samples for bacterial load at hour zero and 16 for this THAI strain experiment. Survival was manually monitored by counting and removing any females not responding to physical stimuli.
