# Supplementary material for: The effects of mating and blood feeding on the immune defense of female Aedes aegypti mosquitoes
Source: PLoS Negl Trop Dis. 2025 Oct 3;19(10):e0013542. doi: 10.1371/journal.pntd.0013542 (PMC12507272; doi:10.1371/journal.pntd.0013542)
Supplement: S1 Fig — (DOCX) [file pntd.0013542.s002.docx]

**Description:** For THAI, we found that unlike LVP, mating did not significantly affect survival rate after a moderate dose infection (p = 0.238, S1A Fig). We also found that mating status did not significantly predict bacterial load (p = 0.316, S1B Fig) or prevalence of infection (p = 0.331, S1C Fig) at this dose.


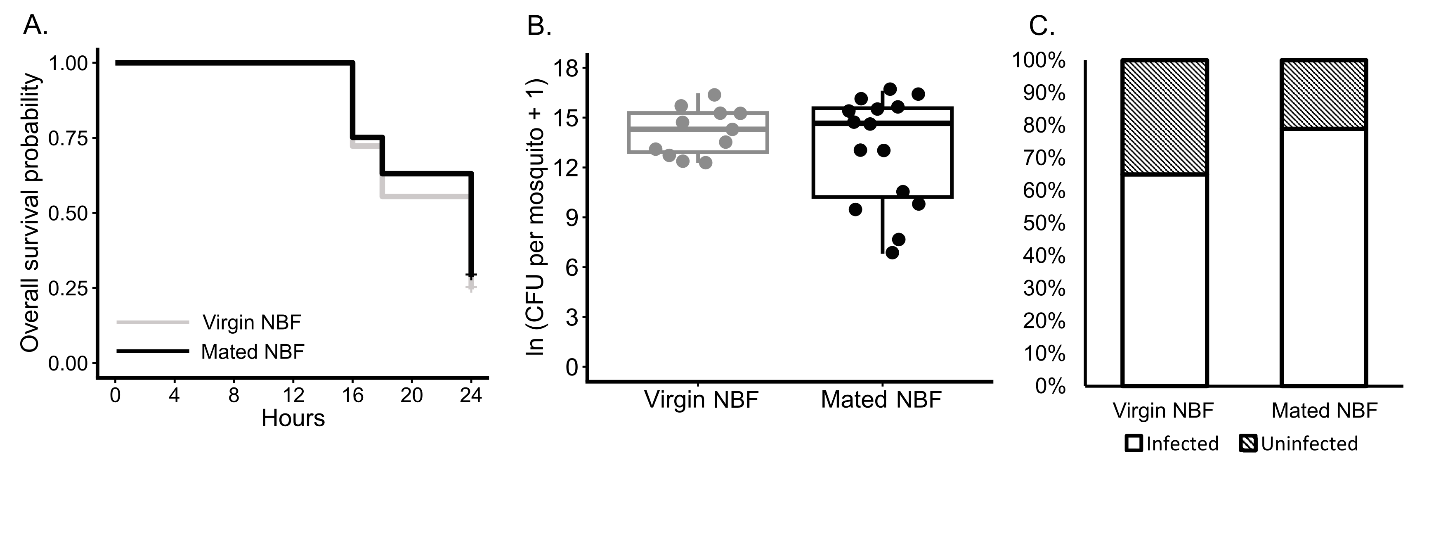
 **S1 Fig. Mating status does not affect female survival or bacterial load in THAI females infected with a moderate dose of *S. marcescens*.** Survival and bacterial load of females injected with 47.18±2.93 CFU of *S. marcescens*. S1A. Survival of virgin (gray) and mated (black) THAI females following infection with S. marcescens (mated n = 157; virgin n = 155; data collected over seven replicate experiments). A Cox proportional hazards model showed no significant effect of mating on survival (p = 0.238). S1B. Bacterial load of virgin (gray) and mated (black) *Ae. aegypti* THAI females at 16 hours post infection (virgin n = 17, mated n = 19; data collected over two replicate experiments). A linear model showed that mating status did not have a significant effect on bacterial load (p = 0.316). S1C. Infection prevalence of THAI females (virgin n = 17, mated n = 19); uninfected is represented by no stripes and infected by black stripes. A GLM showed no significant effect of mating status on infection prevalence post-infection (p = 0.331).
