# Supplementary material for: The effects of mating and blood feeding on the immune defense of female Aedes aegypti mosquitoes
Source: PLoS Negl Trop Dis. 2025 Oct 3;19(10):e0013542. doi: 10.1371/journal.pntd.0013542 (PMC12507272; doi:10.1371/journal.pntd.0013542)
Supplement: S2 Fig — (DOCX) [file pntd.0013542.s003.docx]

**B.**

**A.**

**
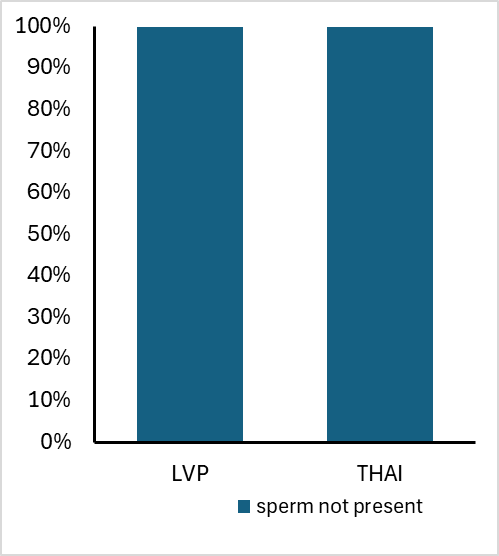
**
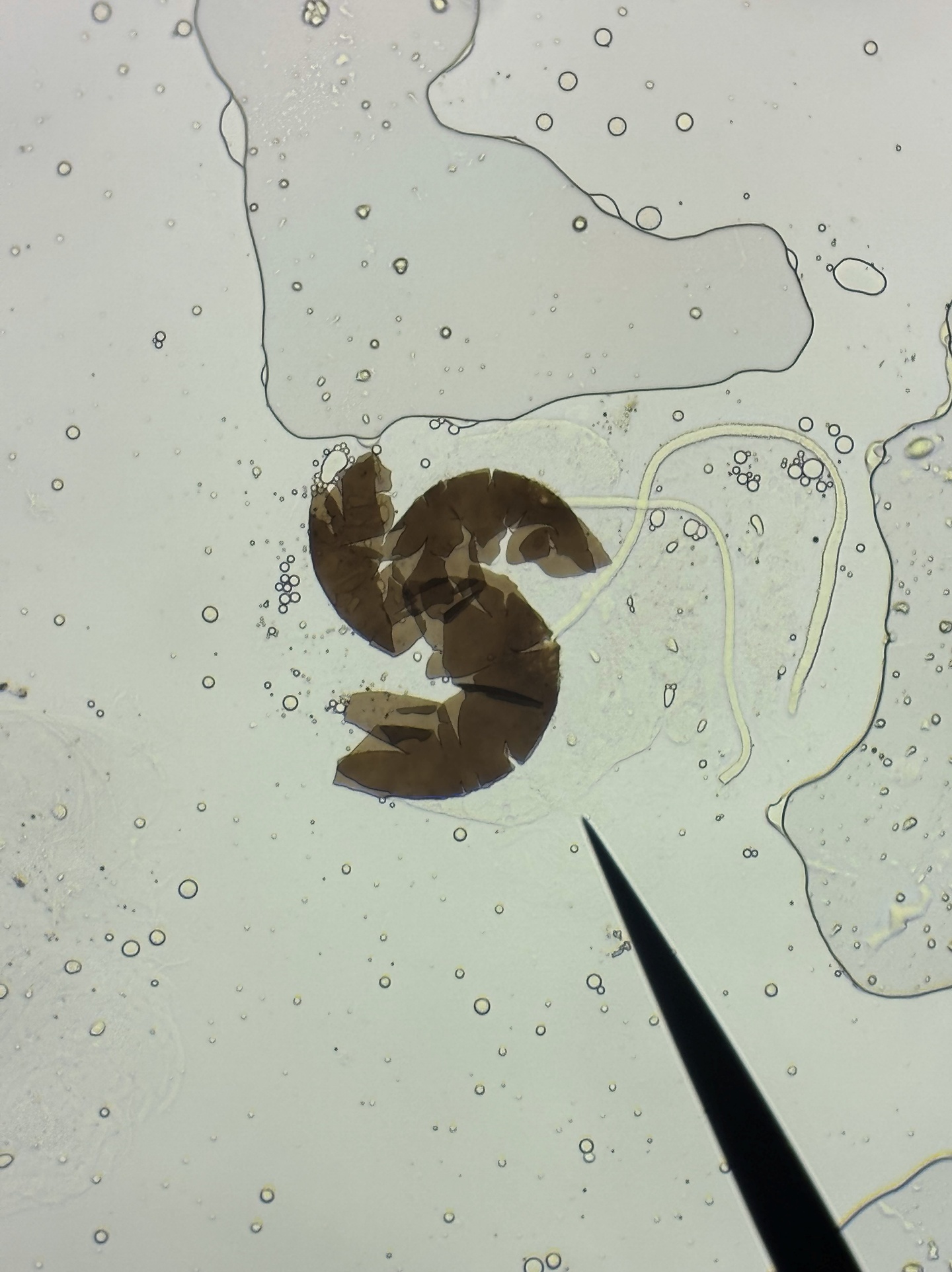


**2**

**1**

**3**

**
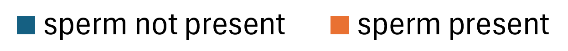
**

**S2 Fig. Females collected no more than 14 hours post eclosion retain virgin status.** Result of spermathecae dissection of LVP and THAI strain females collected no more than 14 hours post-eclosion. S2A. Absence (blue) or presence (orange) of sperm in LVP (n = 83; data collected over three replicate experiments) and THAI (n = 90; data collected over three replicate experiments) strain female spermathecae following eclosion. No sperm was found in any individuals from either strain, resulting in a 100% virgin status for both strains. S2B. Example of a manual spermathecae dissection from a LVP strain female. After the female had been collected (no more than 14 hours post-eclosion), the spermathecae (labeled 1-3) were found to be empty and no sperm was observed.
