# Supplementary material for: The effects of mating and blood feeding on the immune defense of female Aedes aegypti mosquitoes
Source: PLoS Negl Trop Dis. 2025 Oct 3;19(10):e0013542. doi: 10.1371/journal.pntd.0013542 (PMC12507272; doi:10.1371/journal.pntd.0013542)
Supplement: S3 Fig — (DOCX) [file pntd.0013542.s004.docx]

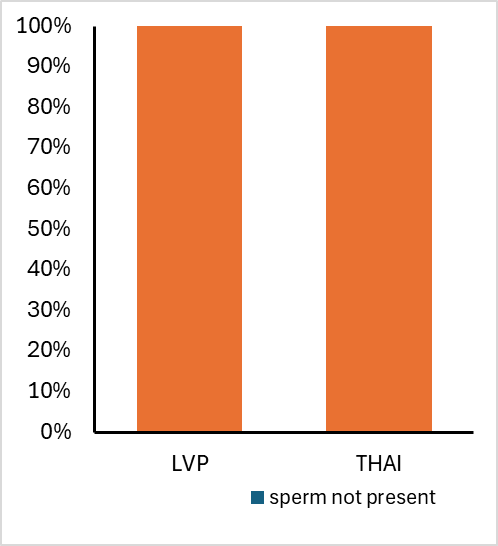
**
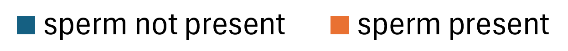
**
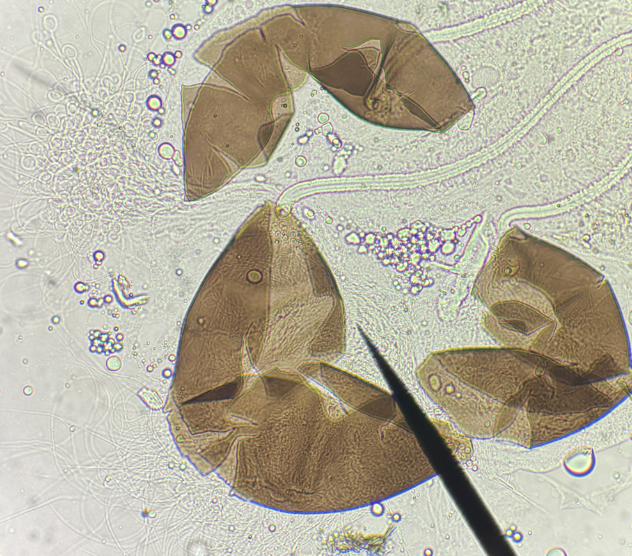


**B.**

**A.**

**S3 Fig. Virgin females allowed to mate with males for a minimum of 32 hours contain sperm.** Result of spermathecae dissection of virgin LVP and THAI strain females allowed to mate for 30-36 and 50-52 hours, respectively. S3A. Absence (blue) or presence (orange) of sperm in LVP (n = 56; data collected over two replicate experiments) and THAI (n = 50; data collected over two replicate experiments) strain female spermathecae following mating. Sperm was identified in all individuals from both strains, resulting in a 100% mated status for both strains. S3B. Example of a manual spermathecae dissection from a LVP strain female. Entangled bunches of hair-like sperm (labeled with red arrows) can be seen spilling out of the burst spermathecae.
