# Supplementary material for: The effects of mating and blood feeding on the immune defense of female Aedes aegypti mosquitoes
Source: PLoS Negl Trop Dis. 2025 Oct 3;19(10):e0013542. doi: 10.1371/journal.pntd.0013542 (PMC12507272; doi:10.1371/journal.pntd.0013542)
Supplement: S2 Code — (DOCX) [file pntd.0013542.s007.docx]

**Fig 2A**

> b7survall <- read.csv(file = "fig2A.csv")

> A <- coxph(Surv(day, status, type=c('right'))~bf*mating+rep, data=b7survall)

> cox.zph(A)

chisq df p

bf 2.609 1 0.11

mating 0.157 1 0.69

rep 2.344 3 0.50

bf:mating 2.260 1 0.13

GLOBAL 5.986 6 0.42

> drop1(A, test= "Chisq")

Single term deletions

Model:

Surv(day, status, type = c("right")) ~ bf * mating + rep

Df AIC LRT Pr(>Chi)

<none> 4790.5

rep 3 4804.4 19.9545 0.0001735 ***

bf:mating 1 4791.9 3.4084 0.0648645

> A1 <- coxph(Surv(day, status, type=c('right'))~bf+mating+rep, data=b7survall)

> cox.zph(A1)

chisq df p

bf 2.9232 1 0.087

mating 0.0554 1 0.814

rep 2.4842 3 0.478

GLOBAL 5.9283 5 0.313

> drop1(A1, test= "Chisq")

Single term deletions

Model:

Surv(day, status, type = c("right")) ~ bf + mating + rep

Df AIC LRT Pr(>Chi)

<none> 4791.9

bf 1 4816.6 26.7428 2.324e-07 ***

mating 1 4798.5 8.5826 0.0033939 **

rep 3 4806.9 21.0485 0.0001029 ***

> #Parsing data by blood feeding status

> #analysis of effect of mating on survival in Blood fed individuals

> AC<- b7survall[b7survall$bf== "B",]

> AC2<- coxph(Surv(day, status, type=c('right'))~mating+rep, data=AC)

> cox.zph(AC2)

chisq df p

mating 0.0288 1 0.87

rep 0.0591 3 1.00

GLOBAL 0.1050 4 1.00

> drop1(AC2, test= "Chisq")

Single term deletions

Model:

Surv(day, status, type = c("right")) ~ mating + rep

Df AIC LRT Pr(>Chi)

<none> 2325.6

mating 1 2324.6 0.9699 0.324715

rep 3 2333.9 14.2146 0.002627 **

> #analysis of effect of mating on survival in Non-Blood fed individuals

> AD<- b7survall[b7survall$bf== "N",]

> AD2<- coxph(Surv(day, status, type=c('right'))~mating+rep, data=AD)

> cox.zph(AD2)

chisq df p

mating 0.407 1 0.52

rep 4.700 3 0.20

GLOBAL 5.748 4 0.22

> drop1(AD2, test= "Chisq")

Single term deletions

Model:

Surv(day, status, type = c("right")) ~ mating + rep

Df AIC LRT Pr(>Chi)

<none> 1867.7

mating 1 1875.2 9.5118 0.002042 **

rep 3 1883.9 22.1981 5.932e-05 ***

**Fig 2B & 2C**

> db<- read.csv(file= "fig2B2C.csv")

> f1<- formula(ceiling(wholeload)~day*mating+rep|day*mating+rep)

> f1A<- hurdle(f1, dist="poisson", link="logit", data= db)

> f1B<- hurdle(f1, dist="negbin", link="logit", data= db)

> lrtest(f1A, f1B)

Likelihood ratio test

Model 1: ceiling(wholeload) ~ day * mating + rep | day * mating + rep

Model 2: ceiling(wholeload) ~ day * mating + rep | day * mating + rep

#Df LogLik Df Chisq Pr(>Chisq)

1 14 -183038

2 15 -249 1 365578 < 2.2e-16 ***

> dbhurdle<- hurdle(f1, dist="negbin", link= "logit", data= db)

> summary(dbhurdle)

Call:

hurdle(formula = f1, data = db, dist = "negbin", link = "logit")

Pearson residuals:

Min 1Q Median 3Q Max

-0.68759 -0.46001 -0.24768 -0.02934 4.53899

Count model coefficients (truncated negbin with log link):

Estimate Std. Error z value Pr(>|z|)

(Intercept) 15.4837 1.3388 11.565 < 2e-16 ***

dayseven -7.0638 1.1413 -6.189 6.05e-10 ***

matingV -8.1749 0.9572 -8.541 < 2e-16 ***

repone -0.1807 1.0567 -0.171 0.86422

repthree -2.5412 1.0224 -2.486 0.01293 *

reptwo -3.9968 1.3838 -2.888 0.00387 **

dayseven:matingV 10.0038 1.4367 6.963 3.33e-12 ***

Log(theta) -0.4956 0.2779 -1.783 0.07457 .

Zero hurdle model coefficients (binomial with logit link):

Estimate Std. Error z value Pr(>|z|)

(Intercept) 1.5200 1.2750 1.192 0.233

dayseven -0.5464 0.9029 -0.605 0.545

matingV 0.8634 1.0358 0.834 0.405

repone -1.6718 1.2139 -1.377 0.168

repthree 0.1966 1.2319 0.160 0.873

reptwo -1.3736 1.2245 -1.122 0.262

dayseven:matingV -2.1959 1.4126 -1.555 0.120

Theta: count = 0.6092

Number of iterations in BFGS optimization: 100

Log-likelihood: -249.4 on 15 Df

> f2<- formula(ceiling(wholeload)~day*mating+rep|day+mating+rep)

> dbhurdle2<- hurdle(f2, dist="negbin", link= "logit", data= db)

> lrtest(dbhurdle, dbhurdle2)

Likelihood ratio test

Model 1: ceiling(wholeload) ~ day * mating + rep | day * mating + rep

Model 2: ceiling(wholeload) ~ day * mating + rep | day + mating + rep

#Df LogLik Df Chisq Pr(>Chisq)

1 15 -249.36

2 14 -250.64 -1 2.565 0.1093

> f3<- formula(ceiling(wholeload)~day*mating+rep|1)

> dbhurdle3<- hurdle(f3, dist="negbin", link= "logit", data= db)

> lrtest(dbhurdle, dbhurdle3)

Likelihood ratio test

Model 1: ceiling(wholeload) ~ day * mating + rep | day * mating + rep

Model 2: ceiling(wholeload) ~ day * mating + rep | 1

#Df LogLik Df Chisq Pr(>Chisq)

1 15 -249.36

2 9 -254.81 -6 10.908 0.09127

> summary(dbhurdle3)

Call:

hurdle(formula = f3, data = db, dist = "negbin", link = "logit")

Pearson residuals:

Min 1Q Median 3Q Max

-0.5319 -0.5043 -0.5013 0.2413 3.5602

Count model coefficients (truncated negbin with log link):

Estimate Std. Error z value Pr(>|z|)

(Intercept) 15.4837 1.3388 11.565 < 2e-16 ***

dayseven -7.0638 1.1413 -6.189 6.05e-10 ***

matingV -8.1749 0.9572 -8.541 < 2e-16 ***

repone -0.1807 1.0567 -0.171 0.86422

repthree -2.5412 1.0224 -2.486 0.01293 *

reptwo -3.9968 1.3838 -2.888 0.00387 **

dayseven:matingV 10.0038 1.4367 6.963 3.33e-12 ***

Log(theta) -0.4956 0.2779 -1.783 0.07457 .

Zero hurdle model coefficients (binomial with logit link):

Estimate Std. Error z value Pr(>|z|)

(Intercept) 0.1335 0.2988 0.447 0.655

Theta: count = 0.6092

Number of iterations in BFGS optimization: 100

Log-likelihood: -254.8 on 9 Df

> f4<- formula(ceiling(wholeload)~day+mating+rep|1)

> dbhurdle4<- hurdle(f4, dist="negbin", link= "logit", data= db)

> lrtest(dbhurdle3, dbhurdle4)

Likelihood ratio test

Model 1: ceiling(wholeload) ~ day * mating + rep | 1

Model 2: ceiling(wholeload) ~ day + mating + rep | 1

#Df LogLik Df Chisq Pr(>Chisq)

1 9 -254.81

2 8 -266.19 -1 22.758 1.838e-06 ***

> #Parsing out significant interaction between mating and day for count data only

> dbcd1<- dbclean[dbclean$day== "one",]

> dbcd7<- dbclean[dbclean$day== "seven",]

> dbcd1y<-lm(log(wholeload)~mating, data= dbcd1)

> drop1(dbcd1y, test= "F")

Single term deletions

Model:

log(wholeload) ~ mating

Df Sum of Sq RSS AIC F value Pr(>F)

<none> 51.714 22.293

mating 1 134.98 186.698 38.266 31.322 0.0001168 ***

> dbcd7y<-lm(log(wholeload)~mating, data= dbcd7)

> drop1(dbcd7y, test= "F")

Single term deletions

Model:

log(wholeload) ~ mating

Df Sum of Sq RSS AIC F value Pr(>F)

<none> 38.209 17.405

mating 1 8.252 46.461 17.360 1.7278 0.2251

**Fig 3**

> hilo <- read.csv(file= "fig3.csv")

> B <- coxph(Surv(hour, status, type=c('right'))~dose*mating+rep, data=hilo)

> cox.zph(B)

chisq df p

dose 0.141 1 0.707

mating 3.676 1 0.055

rep 2.784 2 0.249

dose:mating 1.110 1 0.292

GLOBAL 7.256 5 0.202

> drop1(B, test= "Chisq")

Single term deletions

Model:

Surv(hour, status, type = c("right")) ~ dose * mating + rep

Df AIC LRT Pr(>Chi)

<none> 1683.6

rep 2 1698.7 19.0708 7.225e-05 ***

dose:mating 1 1681.6 0.0296 0.8635

> B1 <- coxph(Surv(hour, status, type=c('right'))~dose+mating+rep, data=hilo)

> cox.zph(B1)

chisq df p

dose 0.132 1 0.716

mating 3.459 1 0.063

rep 2.760 2 0.252

GLOBAL 6.662 4 0.155

> drop1(B1, test= "Chisq")

Single term deletions

Model:

Surv(hour, status, type = c("right")) ~ dose + mating + rep

Df AIC LRT Pr(>Chi)

<none> 1681.6

dose 1 1728.7 49.060 2.482e-12 ***

mating 1 1686.5 6.887 0.008682 **

rep 2 1696.8 19.208 6.747e-05 ***

**Fig 4A**

> b24survall <- read.csv(file = "fig4A.csv")

> C <- coxph(Surv(hour, status, type=c('right'))~bf*mating+rep, data=b24survall)

> cox.zph(C)

chisq df p

bf 0.112 1 0.74

mating 0.116 1 0.73

rep 27.935 3 3.7e-06

bf:mating 0.817 1 0.37

GLOBAL 29.182 6 5.6e-05

> #Replicate does not meet the assumptions of proportional hazards; analysis was completed without replicate

> CP <- coxph(Surv(hour, status, type=c('right'))~bf*mating, data=b24survall)

> cox.zph(CP)

chisq df p

bf 0.154 1 0.69

mating 0.321 1 0.57

bf:mating 1.020 1 0.31

GLOBAL 1.094 3 0.78

> drop1(CP, test= "Chisq")

Single term deletions

Model:

Surv(hour, status, type = c("right")) ~ bf * mating

Df AIC LRT Pr(>Chi)

<none> 1973.5

bf:mating 1 1972.8 1.3191 0.2507

> C1 <- coxph(Surv(hour, status, type=c('right'))~bf+mating, data=b24survall)

> cox.zph(C1)

chisq df p

bf 0.130 1 0.72

mating 0.206 1 0.65

GLOBAL 0.469 2 0.79

> drop1(C1, test= "Chisq")

Single term deletions

Model:

Surv(hour, status, type = c("right")) ~ bf + mating

Df AIC LRT Pr(>Chi)

<none> 1972.8

bf 1 1979.9 9.1032 0.002552 **

mating 1 1970.9 0.0970 0.755512

**Fig 4B**

> b16bacall <- read.csv(file = "fig4B4C.csv")

> b16bacallclean<-b16bacall[b16bacall$wholeload!=0,]

> D <- lm(log(wholeload)~mating*bf+rep,data=b16bacallclean)

> drop1(D, test="F")

Single term deletions

Model:

log(wholeload) ~ mating * bf + rep

Df Sum of Sq RSS AIC F value Pr(>F)

<none> 798.60 131.76

rep 2 6.3125 804.91 128.07 0.1344 0.8747

mating:bf 1 5.7523 804.35 130.05 0.2449 0.6239

> D1 <- lm(log(wholeload)~mating+bf+rep,data=b16bacallclean)

> drop1(D1, test="F")

Single term deletions

Model:

log(wholeload) ~ mating + bf + rep

Df Sum of Sq RSS AIC F value Pr(>F)

<none> 804.35 130.05

mating 1 19.8939 824.24 129.02 0.8656 0.3585

bf 1 9.4887 813.84 128.51 0.4129 0.5247

rep 2 5.7349 810.09 126.33 0.1248 0.8831

**Fig 4C**

> b16bacall <- read.csv(file = "fig4B4C.csv")

> E<-glm(pa~bf*mating+rep, family="binomial", data= b16bacall)

> drop1(E, test= "Chisq")

Single term deletions

Model:

pa ~ bf * mating + rep

Df Deviance AIC LRT Pr(>Chi)

<none> 56.753 68.753

rep 2 58.008 66.008 1.25501 0.5339

bf:mating 1 56.853 66.853 0.10067 0.7510

> E1<-glm(pa~bf+mating+rep, family="binomial", data= b16bacall)

> drop1(E1, test = "Chisq")

Single term deletions

Model:

pa ~ bf + mating + rep

Df Deviance AIC LRT Pr(>Chi)

<none> 56.853 66.853

bf 1 57.691 65.691 0.83802 0.3600

mating 1 57.095 65.095 0.24216 0.6226

rep 2 58.098 64.098 1.24490 0.5366

**Fig S2A**

> ns <- read.csv(file = "figS2A.csv")

> G <- coxph(Surv(hour, status, type=c('right'))~mating+rep, data=ns)

> cox.zph(G)

chisq df p

mating 0.042 1 0.838

rep 13.953 6 0.030

GLOBAL 13.953 7 0.052

> drop1(G, test= "Chisq")

Single term deletions

Model:

Surv(hour, status, type = c("right")) ~ mating + rep

Df AIC LRT Pr(>Chi)

<none> 2397.4

mating 1 2396.8 1.3905 0.2383

rep 6 2394.3 8.9304 0.1775

**Fig S2B**

> bln <- read.csv(file = "figS2BS2C.csv")

> blnclean<-bln[bln$wholeload!=0,]

> H <- lm(log(wholeload)~mating+rep,data=blnclean)

> drop1(H, test= "F")

Single term deletions

Model:

log(wholeload) ~ mating + rep

Df Sum of Sq RSS AIC F value Pr(>F)

<none> 174.85 55.552

mating 1 8.0049 182.86 54.716 1.0530 0.3155

rep 1 0.6829 175.54 53.653 0.0898 0.7671

**Fig S2C**

> bln <- read.csv(file = "figS2BS2C.csv")

> I<-glm(pa~mating+rep, family="binomial", data= bln)

> drop1(I, test= "Chisq")

Single term deletions

Model:

pa ~ mating + rep

Df Deviance AIC LRT Pr(>Chi)

<none> 39.906 45.906

mating 1 40.852 44.852 0.94593 0.3308

rep 1 41.631 45.631 1.72505 0.1890
