# Supplementary material for: The effects of mating and blood feeding on the immune defense of female Aedes aegypti mosquitoes
Source: PLoS Negl Trop Dis. 2025 Oct 3;19(10):e0013542. doi: 10.1371/journal.pntd.0013542 (PMC12507272; doi:10.1371/journal.pntd.0013542)
Supplement: Attachment — Submitted filename: Response to Reviewers.docx [file pntd.0013542.s009.docx]

*** Please note that any line numbers pointed out in our responses are regarding our manuscript with all changes accepted unless stated otherwise***

**Reviewer's Responses to Questions**

**Methods**

**-Are the objectives of the study clearly articulated with a clear testable hypothesis stated?**

**-Is the study design appropriate to address the stated objectives?**

**-Is the population clearly described and appropriate for the hypothesis being tested?**

**-Is the sample size sufficient to ensure adequate power to address the hypothesis being tested?**

**-Were correct statistical analysis used to support conclusions?**

**-Are there concerns about ethical or regulatory requirements being met?**

**Reviewer #1: Even if I consider the topic of interest and scope of the journal, I do not believe the study design was appropriate to address the objectives. As per the stated objectives and article title, the authors intend to study the "immune defense" in the selected strains of mosquitoes. None of the experiments presented actually shows or includes an actual evaluation of the immune effectors in the mosquitoes (AMP expression, hemocyte evaluation, melanization, ROS responses, etc). Measuring survival and infection prevalence are indirect ways to observe the result of an infection, but it does not by itself means you have studied the immune response of the organism.**

Response: We agree with the reviewer that the experiments we conducted do not evaluate immune system activity or immune response per se. We chose to not use the term “immune response” for this reason, as we were most interested in evaluating whole-organism infection outcomes, i.e. bacterial load and survival. We agree that these are not measures of immune system activity, however, we do consider them to be measures of immune defense, in that they reveal the ultimate outcome of pathogenic infection. These terms have been used similarly in other studies which investigate “immunity” by way of *E. coli* clearance in Drosophila (McKean & Nunney, 2001), and “immune response/ disease response” through measuring survival after infection with *S. marcescens* in Gryllus texensis (Shoemaker et al., 2006). While Shoemaker et al. (2006) did measure phenoloxidase (PO) activity, no activity differences were seen between mated or virgin females, but the authors still draw conclusions about immune defense as it pertained to survival post infection. We feel that measures of this nature have inherent value, as they are not limited to measuring elements of immune system activity, which can often be misleading or give contradictory results with regard to overall infection outcomes (Adamo, 2004). Moreover, given that immune defense consists of both infection resistance and infection tolerance (Schneider and Ayres, 2008), it is very likely that mechanisms that govern the bacterial load and/or survival of the infected females in our study fall outside the canonical immune system, thereby underscoring the importance in our opinion of measuring overall infection outcomes. Therefore, while we acknowledge that our study does not include experiments to quantify changes in immune system activity, we still consider the studies we completed sufficient to draw conclusions about the overall immune defense of the female mosquito. To clarify the definition of immune defense and our goals in the paper, we added text on lines 133-134. Additionally, we found a few instances throughout the manuscript where we used terminology that is not consistent with the above definition of immune defense. We modified these instances to prevent confusion on Lines 31-32, 44, 71, 431-432, and 468.

Adamo, SA. How should behavioural ecologists interpret measurements of immunity?, Animal Behaviour, Volume 68, Issue 6, Pages 1443-1449 (2004).

McKean, KA & Nunney, L., Increased sexual activity reduces male immune function in Drosophila melanogaster, Proc. Natl. Acad. Sci. U.S.A. 98 (14) 7904-7909, https://doi.org/10.1073/pnas.131216398 (2001).

Schneider, D., Ayres, J. Two ways to survive infection: what resistance and tolerance can teach us about treating infectious diseases. *Nat Rev Immunol* 8, 889–895 (2008).

Shoemaker, KL.; Parsons, NM.; Adamo, SA. Mating Enhances Parasite Resistance in the Cricket Gryllus Texensis. Animal behaviour, 71 (2), 371–380. https://doi.org/10.1016/j.anbehav.2005.05.007 (2006).

**The population was clearly described, but not necessarily appropriate for**	**the hypothesis being tested. Collecting adult females from cages with**	**males, even if less than 14 hours old, does not guarantee that mating had**	**not occurred. When working with Aedes mosquitoes, it is common practice**	**to separate pupae to allow female mosquitoes emerge without any contact**	**with males, and doing this would have been easier than separating adults.**	**If a statistical sample would have been selected to check the spermatheca**	**contents either by direct observation or by PCR, and absence of mating**	**would have been shown, this would have been also acceptable even if not**	**ideal.**

Response: We thank the reviewer for pointing this out. We did not realize separation at pupation was such common practice and we will use this method in our future experiments. Given that males sexually mature after a 180° rotation of their genitalia after ~15 hours post emergence, and most commonly copulate 20-24 hours post-emergence (Roth, 1948), we assumed it would be sufficient to separate adults within this time frame to ensure virgin status. This approach is further supported by the findings of Ramírez-Sánchez et al., 2023, which showed that females do not mate prior to 22 hrs. post-eclosion. However, we acknowledge that there is natural variation that may occur that we did not account for. Therefore, we have included a supplementary experiment as suggested to ensure that all individuals collected were virgins. In short, we generated LVP and THAI virgin females as described in the manuscript methods, dissected spermathecae from ~30 females from each strain, and observed for the presence of sperm under a compound microscope. This experiment was repeated three times, giving us a total of ~90 females per strain. We found no cases of mated individuals in either strain, verifying our protocol for virgin collection in this manuscript. We have included these results in a supplementary figure (S4).

Ramírez-Sánchez LF, Hernández BJ, Guzmán PA, Alfonso-Parra C, Avila FW. The effects of female age on blood-feeding, insemination, sperm storage, and fertility in the dengue vector mosquito *Aedes aegypti* (Diptera: Culicidae). J Insect Physiol. 2023 Nov;150:104570.

**The justifications for the selection of the mosquito strains used in the study is also not obvious. Why was the THAI strain included only in experiment 3 & 4 and not the others?**

Response: THAI strain individuals were used in our last experiment to examine the effect of mating on immune defense in a strain closer resembling field populations. We agree that clarification is needed, and that we failed to make this clear to readers. To mitigate confusion and improve the flow of the manuscript, we have moved our THAI strain experiment to supplementary material (S1: methods; S2: figures and results), leaving the main manuscript fully based on LVP strain experiments. We have also included a reason for inclusion of this THAI strain experiment in lines 153-156 & 494-496 and discussion of the caveats it raises in lines 491-496. It should be noted that after closer examination of our dosing data, we decided to consider our THAI experiment to be a moderate dose experiment rather than a low dose experiment- this has been explained in our response to reviewer #3 under “editorial and data presentation modifications; Page 9, line 191.”

**Was there any indication that any of the variables to be studied (i.e. immune responses, mating, longevity, etc.) could potentially be different between strains? A reader with knowledge about the THAI mosquito strain would know that this colony has been supplemented annually with F1 eggs from the field, and therefore bares more resemblance to field strains than the LVP strain. Yet, this is not mentioned anywhere in the manuscript and therefore we don't know if this was the reason to include this strain in the study or not.**

Response: We agree with the confusion that this causes the reader and have therefore moved THAI strain figures and results to supplementary files as described above. We have included lines 153-156 to make it clear to readers without this knowledge that THAI strain individuals are more comparable to field populations than LVP.

**Sample size was sufficient for the experiments presented and I find no ethical concerns with the work.**

**Reviewer #2:**

**1.The terms “bacterial load” and “infection prevalence” appear repeatedly across multiple sections and are somewhat redundant in meaning. We recommend merging or simplifying these terms for clarity. Additionally, many sentences in the Introduction are overly nested; these should be revised for conciseness.**

Response: The phrases “bacterial load” and “infection prevalence” refer to unique measurements. Bacterial load refers to the quantity of bacteria in each individual sample. Infection prevalence refers to the proportion of individuals testing positive for infection (regardless of bacterial load) in a group. As these are different measurements, we believe using different terms to describe them is necessary. We have directly defined these terms in lines 146-148 to clarify these meanings.

**2.The number of keywords is excessive; the authors should consider reducing and refining them.**

Response: We will reduce the number of keywords.

**3.As Aedes aegypti is a primary vector for yellow fever virus, the authors should clarify why they focused on bacterial infection instead of the effects on viral transmission (e.g., dengue or Zika virus).**

Response: This has been clarified in the introduction in lines 125-129.

**4.The Introduction does not explain the rationale behind choosing Serratia marcescens as the bacterial pathogen. The authors should provide justification for this selection.**

Response: This has been clarified in the introduction in lines 135-137.

**Furthermore, the rationale for selecting the high, medium, and low infection doses requires support from relevant literature.**

Response: We agree with the reviewer that our rationale for selecting particular doses is not explained. Our doses were selected through preliminary survival trials in LVP individuals and were chosen based on observed survival rates of individuals. The “high” dose caused very high mortality (i.e. >80% on average by 24 hrs.) and the moderate and low doses contain, on average, 39% and 86% fewer bacteria than the high dose, respectively. The nomenclature of "high, moderate, and low” dose was chosen post hoc, where the values are expressed relative to one another. While relative, we consider the terms to be useful shorthand and to accurately reflect the dosing data. We report the accurate CFU/mosquito values for each dose for full transparency and report all dose data in supplementary file S8. Moreover, we note that our survival results between doses supports the use of these different terms, in that the high dose caused high mortality, moderate caused moderate mortality, and low dose the lowest mortality.

**Reviewer #3:**

**1. It is not clear why the authors used two different strains of Aedes aegypti. Specifically, why was one strain (THAI) only used for one of the experiments.**

Response: We chose to examine a moderate dose infection (previously considered a low dose) in THAI strain females in order to investigate effects in a strain that more closely resembles a field strain. We have clarified this in manuscript lines 153-156. Additionally, we have moved our THAI experiment and methods to supplementary files S2 and S1, respectively, to mitigate any further confusion for readers.

**2. The description of the experimental design is confusing. It would be helpful to have a experimental design figure to help clarify the differences between the different experiments.**

Response: We understand that the experimental design may be confusing to readers. We have moved all THAI strain methods to supplementary files S1 and S2, respectively, and have included an experimental design figure for LVP strain experiments as figure 1.

**________________________________________**

**Results**

**-Does the analysis presented match the analysis plan?**

**-Are the results clearly and completely presented?**

**-Are the figures (Tables, Images) of sufficient quality for clarity?**

**Reviewer #1:**

**The results are clearly presented. The figures that present survival data should include the CI.**

Response: We agree and understand that the inclusion of confidence intervals is valuable for the reader. However, we found that adding the CI for each survival curve (regardless of color or opacity changes) caused graphs to be very difficult to visually parse, which effectively negates any benefit from including the CI for each curve. This is likely due to the tightness/closeness of the plotted curves and the fact that we have multiple treatments on a single graph. We find that leaving these survival plots as they are provides substantial information for the reader without making the graphs muddy/ difficult to interpret. We note that our statistical analysis incorporates the variation in the dataset that would be reflected by plotting the CI, so our plots coupled with the analysis results sufficiently evaluate significance.

**I am concerned with the number of data points presented in figure 1B, considering the number of mosquitoes included, and the number of replicates that the authors made, there should be more points in the 7 day virgin NBF group.**

Response: We agree that having more data points here would have been ideal, however, due to higher mortality among virgin females (and previous sampling at day 1), there were generally fewer virgin individuals to sample at day 7. Moreover, for the individuals we did sample, few had detectable infections on day 7 (see Fig. 2C). For these reasons, we have a low sample number of bacterial load counts for the 7 day virgin NBF group, despite our sample sizes being similar between all treatments (9-13 individuals per treatment group). This is reflected in our included raw data supplementary file (S5).

**Reviewer #2:**

**1.The authors are encouraged to assess changes in key immune markers within the mosquito system, such as antimicrobial peptide (AMP) expression or Toll/IMD signaling pathways.**

Response: We agree with the reviewer that the aforementioned experiments would add value and insight to our project. However, we were most interested in evaluating whole-organism infection outcomes, i.e. bacterial load and survival, and therefore chose to measure them rather than the immune markers listed here. We acknowledge that bacterial load and survival are not direct measures of immune system activity, however, we do consider them to be measures of immune defense, in that they reveal the ultimate outcome of pathogenic infection. We feel that measures of this nature have inherent value, as they are not measuring elements of immune system activity, which can often be misleading or give contradictory results with regard to overall infection outcomes (Adamo, 2004). Moreover, given that immune defense consists of both infection resistance and infection tolerance (Schneider and Ayres, 2008), it is very likely that mechanisms that govern the bacterial load and/or survival of the infected females in our study fall outside the canonical immune system, thereby underscoring the importance in our opinion of measuring overall infection outcomes. Therefore, while we acknowledge that our study does not include experiments to quantify changes in immune system activity, we still consider the studies we completed sufficient to draw conclusions about the overall immune defense of the female mosquito. We agree that future studies investigating the mechanism of our findings and immune system activity in particular would be valuable, but we consider them beyond the scope of this study, in which we strove to measure overall outcomes of infection.

Adamo, SA. How should behavioural ecologists interpret measurements of immunity?, Animal Behaviour, Volume 68, Issue 6, Pages 1443-1449 (2004).

Schneider, D., Ayres, J. Two ways to survive infection: what resistance and tolerance can teach us about treating infectious diseases. *Nat Rev Immunol* 8, 889–895 (2008).

**2.The claim that blood feeding impairs immune defense is attributed to egg production or hormonal fluctuations, but these explanations lack direct experimental evidence.**

We were careful to use language to indicate that these hypotheses are untested (e.g. “It is possible that...” line 452) to ensure that it was clear to the reader that these remain open questions. While we acknowledge that these hypotheses lack experimental evidence, we feel we provided adequate context to support the logic behind the suggestion. Moreover, we consider some moderate speculation and interpretation to be appropriate for a discussion section given appropriate context and support from the literature.

**3.All treatment groups were injected with S. marcescens, and no uninfected (healthy) control group was included. The dose ranges used (30.9±11.8 to 184.6±36 CFU) are quite wide, and there is considerable variability in actual doses delivered. Only the immune responses within a 12–48 hour window post-injection were assessed, leaving early or late-phase immune responses unexamined.**

Response: We completed sterile injury controls when completing the experiments but did not initially include these data. We have now included them, as described in lines 227-231. We have added a brief explanation of the effects of sterile needle injury on survival in the same lines. Regarding the window in which we chose to assess survival and bacterial load, this was chosen based on the findings of our first experiment. In our first experiment (see fig. 2), we observed survival over the course of 7 days. This extended period of time (compared to the following experiments) was much longer, in order for us to pinpoint times at which mortality occurred. After observing that nearly all mortality took place within the first ~24 hours, we decided to complete the rest of our experiments within this time frame. Please refer to our response to Reviewer #3 (Page 9, line 191 “Editorial and Data presentation modifications”) for a response to the concern of dose ranges.

**4.Flow cytometric analysis of hemocyte proliferation should be included, and critical immune indicators such as antimicrobial peptides and phenoloxidase were not evaluated.**

Response: Please see our response to comment #1 in this section, where we discuss this issue.

**5.The term “significant” is frequently used without providing exact p-values or confidence intervals, making the results interpretation vague.**

Response: We checked the manuscript for any instances of this nature and removed one on line 371 (in our manuscript submission including tracked changes). We acknowledge that we did not include a p-value in one of our statements explaining figure 2C (line 360 [document including tracked changes]): “Presence/absence data analysis revealed no significant effects of mating, day, or the interaction between these factors on the prevalence of infection (fig. 21C).” This is due to the nature of our hurdle analysis, in which we performed a backwards elimination approach, where all non-significant variables were removed and the simplified model was compared to the full model. This revealed no significant different difference between the models, indicating none of the removed variables were significant, but did not generate individual p-values. We also acknowledge that we use the term “significantly” a few times in the discussion, but this is in reference to previous uses in the results in which proper statical analysis results were provided.

**6.The Results section should be divided into clearly structured subsections to improve the logical flow of the manuscript.**

Response: Sub headers have been added for clarity.

**7.Authors should be required to submit color versions of the figures for better clarity.**

Response: While we understand and appreciate that coloured graphs may be more visually appealing for readers to look at, we do not feel that the included figures are less clear in their current state. All graphs include figure legends addressing colouration or group descriptions in x-axis labels where applicable. We find that the use of Black/grey/white also appeals to colour blind readers, and that no understanding is lost from not including colour. However, we acknowledge that the colouration of our presence/absence plots may be misleading for the reader since the colours are the same as our bacterial load/survival plots. We have changed these presence/absence plots to include patterns that are unique from our other graphs for better clarity.

**8.Statistical significance indicators (e.g., p-values, asterisks, confidence intervals) are missing from some of the figures and should be added for transparency.**

Response: We agree that p-values should be added to our high dose infection bacterial load graph (figure 2B), as a significant effect of mating was found for day one but not for day seven. We have indicated p-values directly on the graph for clarity. We have clarified our reasoning for not including significance indicators elsewhere in our response to reviewer #3, point 1.

**Reviewer #3:**

**1. For all figures, significant differences should be made clear with asterisks.**

Response: We agree and have added asterisks to our figure 2B bacterial load plot for clarity. However, given the nature of most of our statistical analyses and the use of multiple predictive factors throughout, we find that adding asterisks will add confusion and could potentially be misleading by highlighting one aspect of the analysis at the expense of the other. For example, in figure 4B (previously figure 3B), our graph follows both blood feeding and mating status. Because we are examining these terms individually as well as their interaction, we feel that asterisks are not appropriate, as they would necessarily highlight either the effect of mating or the effect of blood feeding, and not both, and may detract from the full findings of the model. We find that the summary explanation of the statistical evidence we provide in our figure legends and results section allows the reader to understand statistical differences in full.

**2. Fig. 1C and Fig. 3C & F The exact sample sizes with numerator and denominator should be included in the figure or legend.**

Response: These sample sizes have been added in the figure legends. Please note that Fig, 3C and F are now found in supplementary file S2 as figure S2A and S2C.

**3. The figure legends in general rehash what was written in the results section. The figures should be labeled well enough to be self-explanatory, but the legend should be there to help explain the figure. The figure legends should be re-written so as to not re-state the findings but rather explain the figure.**

Response: We feel that our figure legends do help to explain figures, as we have included details on the experiment performed, description of experimental group/legend colouration, the type of statistical analysis done, and p-values (including interactions). We find that our figure legends function as stand-alone descriptors of our graphs and wish to maintain this common stylistic approach. We have used this approach in previous publications as well, and in case it provides useful context, please see the reference below for an example of a recent publication.

El-Dougdoug, Noha K et al. “An obligate microsporidian parasite modulates defense against opportunistic bacterial infection in the yellow fever mosquito, Aedes aegypti.” mSphere vol. 9,2 (2024): e0067823. doi:10.1128/msphere.00678-23

**4. Ln. 327 The word prevalence is repeated and one should be removed.**

Response: This has been edited.

**5. Ln. 267 and Fig. 1 legend the authors state one of their analyses was marginally significant. It’s either significant or not. Marginally should be removed as a descriptor.**

Response: We have removed this term.

**________________________________________**

**Conclusions**

**-Are the conclusions supported by the data presented?**

**-Are the limitations of analysis clearly described?**

**-Do the authors discuss how these data can be helpful to advance our understanding of the topic under study?**

**-Is public health relevance addressed?**

**Reviewer #1:**

**Based on the comments made in the methods section, I do not believe the conclusions are supported by the data presented. The absence of any actual evaluation of the immune effectors of the mosquitoes impairs drawing a conclusion about "immune defenses" being affected or not by mating. Mere survival and infection prevalence are not sufficient to make this statement. The title also ends up being misleading, as it not only indicates that mating affects the immune defense, but that blood feeding also does it, when most of the authors own results end up showing that blood feeding in "vir" vs mated individuals have not difference.**

Response: We have addressed this concern by the reviewer in our responses to this reviewer’s first comment above. With regard to the last sentence of this comment, our title indicates that blood feeding (irrespective of mating status) impacts immune defense, which is supported broadly by our findings (e.g. the main effect of blood feeding on survival in Fig. 2 and Fig. 4).

**Reviewer #2: (No Response)**

**Reviewer #3:**

**1. The authors find that mating leads to increased survivorship yet higher bacterial counts with very significant differences. These seems counter intuitive yet they did not discuss this discrepancy at all. Further, the authors suggest that mating could bolster the immune response yet if this were the case then you would expect reduced bacterial counts. The authors did not provide any evidence that the immune response was altered after mating. To make such claims additional data examining the expression levels of the Toll/ IMD pathways and/ or antimicrobial peptides would be needed. This also applies to the title. They suggest immune defenses in the title but no data directly supporting such claims.**

Response: We greatly appreciate this input. Please view our response above to Reviewer #1 under “methods” for a clear response to this concern regarding the term “immune response.” To clarify the definition of immune defense and our goals in the paper, we added text on lines 133-134. We modified all instances in which our terminology was not consistent to prevent confusion on lines 31-32, 44, 71, 431-432, and 468.

We acknowledge that we did not initially provide any discussion of the discrepancy around increased survivorship/ higher bacterial counts. We have included lines 404-410 in our edited manuscript discussion to acknowledge that our results are similar to those found in Reitmayer et al. (2021), and that our results indicate mating may have an effect on infection tolerance (but that more research is needed to confirm this).

**2. Ln. 397 Starting a paragraph with “For example” is kind of odd. I would suggest re-writing this transition.**

Response: We have made this change. See line 430.

**________________________________________**

**Editorial and Data Presentation Modifications?**

**Use this section for editorial suggestions as well as relatively minor modifications of existing data that would enhance clarity. If the only modifications needed are minor and/or editorial, you may wish to recommend “Minor Revision” or “Accept”.**

**Reviewer #1:**

**Page 3, line 58 – It is stated that mating affects lifespan, does it increases it? Or reduces it? Just by the sentence this is not clear.**

Response: This has been clarified in the introduction in line 58.

**Page 3, line 59 – but non IN no NBF individuals.**

Response: This has been edited.

**Page 3, line 60-61 – The reference “Change et al., 2021” is not included in the bibliography. ChatGpT?**

Response: This has been changed to “Chang et al. 2021.” We did not use chat GPT for any part of this manuscript.

**And there are several possible articles to be cited on this topic that have not been included as reference for this statement.**

Response: We have an added additional reference in this line to back up this statement.

**Page 3, line 62—An article whose primary focus is the effects of mating on the immune system could expand a little bit on the literature about the topic in the introduction, especially because in this sentence it is stated that the subject is “many other insects”, citing a single review (Oku et al. 2019) for this seems a little bit lazy.**

Response: We appreciate the reviewer’s concern. We have removed this sentence during re-writing and have instead included this review in a more comprehensive list of relevant citations on line 74.

**Page 3, line 64 – If you consider previous work published in Aedes aegypti in mating effects over immunity, including but not limited to: Alfonso-Parra, 2016; Camargo, 2020; Meng-Meng Chang, 2021; Reitmayer, 2021; League, 2021; Taracena-Agarwal, 2024; I would say that the mating effect on immune defense in Ae. Aegypti is not “largely unexplored”. Rather, the mechanisms determining the immune modulation caused by the different components of the mating event need to be better understood.**

Response: We agree with the reviewer and appreciate the comment. We have included Reitmayer, 2021 in lines 68 and 404, Alfonso-Parra, 2016 in lines 64-65, and a second citation for Taracena-Agarwal, 2024 in line 62.

After careful review, we feel that Chang, 2021 is relevant to our description of the immunosuppressive nature of JH, but it is less well-suited for our discussion on the effects of mating on immune defenses because the effect of mating per se was not measured. Chang, 2021 has been cited in lines 425. While League, 2021 does address blood feeding, we do not feel that this paper fits into our discussion about the effects of mating on immunity given that it does not include any measures of immunity. We have therefore chosen to omit it from this discussion as well. Camargo, 2020 does identify one group of genes (cytochrome P450s) which are thought to be immunosuppressive (for sperm protection). However, because very few immune-related genes were described in this study and those that were have an indirect connection to immunity, we opted to exclude this reference.

**Page 4, line 77 – reference is needed right after “post-mating”.**

Response: A citation has been inserted here.

**Page 5, line 96 – if a careful literature review is done, this sentence would not be included in the manuscript, mating impact in systemic immune defense has clearly already been investigated in Ae. Aegypti.**

Response: Our language was not clear here – We agree that removing this sentence is best and does not disrupt the literary flow of this section.

**Page 6, line 117 – no need to cite the same reference in two consecutive sentences.**

Response: This has been edited to remove the references in the second sentence.

**Page 6, line 122 – I believe it is important to add that you studied these conditions in “young” females, at a “short” time of exposure between the events, with varying doses of Serratia.**

Response: this line has been edited to include the age of the females at the time of treatment and it also mentions the varying doses of Serratia. The time of exposure between the events is indicated in the methods experimental design lines 142-144, the general methods line 172, 183, 217, and in the figure 1 legend.

**Page 6, 128 – This section needs work to be complete as a “experimental design” outline. For the 4 types of experiments performed, it would be useful if you would first denominate the function or intent of each experiment before detailing it. Like: High dose experiment, low dose experiment, blood feeding experiments, etc. Then, it is necessary to add the ages the mosquitoes had, times for the mating, when the injections were performed (and the reasoning behind the selection of these times), and how long after injection the blood meals were performed. It is also not clear to me why only experiment 4 includes the THAI strain; this should be explained as part of the experimental design. Why the larval densities were different in experiment 4 should also be explained.**

Response: We have included the intent of each experiment as sub headers throughout the results, and agree that this more clearly organizes the flow of our experiments. We have also included a timeline in the experimental design legend including age at which females were mated, blood fed, and infected.

We have moved our THAI strain experiment to a supplementary file S2 to avoid confusion for the reader. We have also included a statement in the experimental design explaining our choice to run an experiment in THAI (to examine the effect of mating on survival and bacterial load in individuals more closely resembling field populations).

**Page 7, line 134 – Changes in mosquito larval densities can induce significant changes in adults' nutrient reserves. As it happens in lower density trays, mosquitoes reared with more food availability can live longer, endure more environmental pressures, or potentially be more fit for mating. If there were no changes in these parameters with the changes in rearing conditions, this information should be added as supplemental information. If the tray size, water volume, and food mass were adjusted to be kept stable through the change in the number of larvae per tray, this should also be written in the methods.**

Response: We agree that larval densities along with other environmental factors such as bacterial presence can affect adult nutritional reserves and adult life history traits and appreciate the reviewer for raising this concern. Because larvae ingest both bacteria and non-living organic matter, and both can affect larval and adult traits, they may both be viewed as forms of “nutrition.” We find that if we controlled for nutrition by way of food mass (cat food was used in our study), that we would also need to control for bacteria (genera and quantity) in each tray. Because our study did not focus on the effects of larval nutrition, we chose to control nutrition by adding the same amount of food to each larval tray during experimental setup. Pupae were collected from all trays in the same 2-day window, which ensured that the same number of individuals used which were reared in an “unstable” environment was matched across experiments. To avoid any confusion regarding our THAI experiment, we have moved this experiment (methods and graphs) to supplementary files S1 and S2, respectively. No other edits were made in the manuscript as we did not make adjustments to food mass, water volume, or bacteria per tray during the course of any experiment.

**Page 7, line 150 – what Roth describes in the 1948 paper is that males are not interested in “very young females”, 2-3 hours old, and even with these females they did attempt to mate on a small percentage of success. This reference also describes that it takes less than a minute to mate and other great many details of the mating event. Additionally, it is well established that males emerge earlier than female pupae, by times ranging up to 2 days, so it is mostly always that females will encounter mature males upon emergence. With all of this in mind, the protocol in which the “virgin” mosquitoes were selected is not really one that can guarantee the absence of mating. Collecting virgin adults from a cage, after 12 or 14 hours will very probably have a very significant percentage of mated females already. Therefore, and without any confirmation of mating status by the authors (either by direct observation of the spermathecae or by PCR), I can not say I trust their selection of “virgin” individuals and makes it hard to trust the data corresponding to this group in the manuscript.**

Response: We have included a virgin status experiment for both LVP strain individuals and THAI strain individuals (THAI strain experiments have been moved to supplementary files S1 and S2) to verify the methods for our virgin collections. The results of this experiment have been included in supplementary file S3. In short, we replicated egg hatching and adult collection methods described in our methods. We checked pupae every 12-14 hours, and separated all eclosed mosquitoes by sex and collected adult females into cages during each of these checks. We then dissected out spermathecae from ~30 virgin females and examined them for the presence of sperm. After three replicate experiments per strain, we found that all three spermathecae in all females were empty, i.e. contained no sperm. This was true for both LVP (total n = 83) and THAI (total n = 90) individuals. We find that the lack of sperm in all of these individuals validates the methods for virgin collection used in our main experiments. We also note that female virgin status for the first ~22 hrs post eclosion has been further validated by Ramírez-Sánchez et al., 2023, and we have added this citation to the manuscript (line 168).

Ramírez-Sánchez LF, Hernández BJ, Guzmán PA, Alfonso-Parra C, Avila FW. The effects of female age on blood-feeding, insemination, sperm storage, and fertility in the dengue vector mosquito *Aedes aegypti* (Diptera: Culicidae). J Insect Physiol. 2023 Nov;150:104570.

**Page 7, line 156 – regarding the mating procedure, this is also not an ideal protocol for a study of “mating effects”, which have a known temporal evolution. If the mating is just “assumed” by the mere presence of males being placed in the cage, some females could be mated immediately after the exposure and other could be mated over a day and a half or more later (depending on their experiment number), and at the dissection time there is no way of knowing which female just copulated and which did it a day ago. In this particular experimental design, I even wonder if the females were already mated and could have been refractory by the time of this selected exposure with the males.**

Response: We agree that combining males and females into one large cage does not allow us to identify exactly when each individual has copulated. We modeled our methods after Villarreal et al. (2018) in which they mixed males and females into one cage for 48 hrs to produce mated females. We completed an additional mating verification experiment (as described below) to verify that the amount of time we allowed males and females to mate in this setting was sufficient to guarantee mating occurred (LVP:30-36 hours; THAI: 50-52 hours). While we do not know exactly at what time point each female mated, it has previously been shown that when *Ae. Aegypti* males and 4-day-old females are mixed into a cage together for only two hours, 99% of all females mate (Ramírez-Sánchez et al., 2023). Additionally, when *Ae. aegypti* females are introduced to males individually, they typically require 10-15 minutes to mate, with few pairs requiring ~30 minutes (Mayilsamy et al., 2024). Therefore, it is extremely likely that all females in our experiment mated within a similar window of time. We feel that this information, paired with our mating verification experiment, supports our choice of methods.

Mayilsamy, M., Subramani, S., Veeramanoharan, R. et al. Mating of unfed, engorged, and partially to fully gravid Aedes aegypti (Diptera: Culicidae) female mosquitoes in producing viable eggs. Parasites Vectors 17, 362 (2024). https://doi.org/10.1186/s13071-024-06433-z

Ramírez-Sánchez LF, Hernández BJ, Guzmán PA, Alfonso-Parra C, Avila FW. The effects of female age on blood-feeding, insemination, sperm storage, and fertility in the dengue vector mosquito Aedes aegypti (Diptera: Culicidae). Journal of Insect Physiology. 2023;150: 104570. doi:10.1016/j.jinsphys.2023.104570

Villarreal, Susan M et al. “Male contributions during mating increase female survival in the disease vector mosquito Aedes aegypti.” Journal of insect physiology vol. 108 (2018): 1-9. doi:10.1016/j.jinsphys.2018.05.001

**In the case where the mating event is the main variable, investing an hour or so to observe the cages while the males are introduced and carefully aspirating the females you actually see copulating is the best guarantee that you have synchronously selected mated females for your study. This, or at the time of dissection, saves the spermathecae of the individuals and confirms sperm presence.**

Response: We agree that observing copulation directly would ensure that females mated. To verify our procedure and show readers that our method of mating (placing males and females into the same cage for 30-36 hours) results in 100% mated females, we have conducted a mating verification experiment and have included the results of this experiment in supplementary file S4. While we have moved all THAI strain experiments out of the main manuscript to supplementary files S1 and 2, we conducted a mating verification experiment for this strain as well. We have included details of these experiments (methods and reasoning) in the manuscript on lines 189-194. In short, we hatched eggs, collected virgins (for which the method was also verified through an additional experiment included in supplementary figure S3), allowed individuals to age, and mated individuals in the exact same way as described in our methods (for LVP and THAI individuals). After LVP and THAI individuals were allowed to mate for 30-36 hours and 50-52 hours, respectively, males were discarded, and females were placed in clean 1L bucket cages. No more than 3 days after separating out females, we dissected out the spermathecae of each female and examined them for the presence of sperm. We found that in 50 THAI individuals (collected over two replicates) and in 56 LVP individuals (collected over two replicates), all individuals from both strains presented with sperm in at least one spermathecae.

**Page 8, line 171 – It should be defined if the females removed were only the ones that did not eat at all and partially engorged females were included in the study or not.**

Response: This has been clarified in the methods in lines 205-207.

**Page 9, line 191 – Even in the Khalil paper, where the methodology is described, it is stated and discussed that the flaw in the method is that it does not allow precise control of the inoculated bacterial load. I am having trouble understanding why experiments 1 to 3 were pricked and the experiment with THAI was injected. Seems to me, that having access to a Nanoject II, there is no obvious reason as of why some experiments would be done with one method over the other. It would be nice to have an explanation of this over in the experimental design section.**

Response: We agree that this difference in methodology may be confusing to readers and greatly appreciate this input. We have moved all THAI strain experiments and methods to supplementary files S1 and S2 to mitigate some of this confusion.

We acknowledge that our moderate and high dose pinprick infections include larger margins of error and have therefore included a formal evaluation of our dosing to improve accuracy and clarity. To account for variability seen through pinprick infections “we quantified the average infectious dose for each experiment after removal of outliers (identified as points more than 1.5 times above or below the interquartile range)” and have explicitly expressed this in the manuscript in lines 237-241.

For convenience to the reviewer and to facilitate understanding of the following explanation, doses are summarized here:

High dose (fig. 2): 113.16 ± 15.9 CFU

High dose (formerly Moderate dose 2) (fig. 3): 108.64 ± 13.3 CFU

Moderate dose (formerly Moderate dose 1) (fig. 3): 42.33±9.76 CFU

Moderate dose (formerly Low dose) (THAI strain, file S2): 47.18±2.93 CFU

Low dose (fig. 3): 9.38 ± 1.2 CFU

Upon further consideration of the dosing data, we determined that our “moderate dose 2” in fig. 3 should rather be considered a “high dose” given that the higher dose in fig. 3 is similar to the high dose used in fig. 2 (108.64 ± 13.3 and 113.16 ± 15.9 CFU, respectively) and that the survival rate of infected individuals was quite similar to our “high dose” experiment in fig. 2. After completing a Welch’s T-test, we also determined that the doses in fig. 3 were significantly different from each other (p = 0.000203) and opted to therefore modify our description of dosing in fig. 3 to be a high dose vs. a moderate dose. We have edited this throughout the manuscript and have changed the figure/ experimental name to “high vs. moderate dose”. All dosing data, and the abovementioned T-test results (before and after outliers were removed) can be found in supplementary file S8.

After removal of outliers from all dosing data, we also found that our THAI experiment depicted both CFU count and survival rates much closer to our (newly named) moderate dose (47.18±2.93 CFU compared to 42.33±9.76 CFU, respectively). Therefore, we have corrected the presentation of our THAI experiment to a “moderate dose THAI” experiment and have indicated this throughout the manuscript. We also discussed caveats raised by the findings using THAI strain in lines 491-496. While we acknowledge that using a different injection method for our THAI females may be confusing, we used a Nanoject here to control for variable dosing seen in our LVP experiment. We hope that these changes in addition to our inclusion of a supplementary dosing file mitigate these concerns.

**Page 9, line 207 – I understand there were experiments done where infected females were blood fed after the infection. It should be presented here, what was the timeline for this series. The authors should state at what age mosquitoes were mated, when they were infected, and then at what age they were blood-fed. If injections affected the volumes of blood acquired, this should also be recorded to guarantee that the differences observed were not actually due to variations in the quantities of blood acquired.**

Response: This line refers to our THAI strain experiment which did not include any blood feeding. These THAI experiments including all methods have been moved to supplementary files S1 and S2 to avoid confusion for the reader. For LVP experiments, which did include blood-feeding, we included the ages at which they were mated, blood fed, and infected in the methods experimental design lines 142-144, the general methods line 172, 183, 217, and in the figure 1 legend. No experiments included blood feeding post-infection, and we apologize for the confusion. We hope that including the aforementioned timepoints and general experimental overview (fig. 1) clarifies this confusion.

**Page 12, line 264 – confidence intervals for the data on figure 1A should be added to the figure. These would be especially important for understanding the “marginally significant” interaction found.**

Response: We acknowledge that CIs are useful data to include, particularly to visually explain the term “marginally significant”. However, when creating our graphs, adding CIs to survival plots made the graphs nearly illegible (Likely due to the closeness of some of the lines in the graph). We feel that adding the CI will make this graph much more difficult to visually interpret, regardless of whether colour is added or the opacity of the CIs are changed. Our statistical analyses properly evaluate each factor of interest and have been included to allow the reader to clearly interpret the data.

We agree that the term “marginally significant” may be confusing to the reader and that we should be direct in our statistical conclusions. We have removed this phrase where it was present (lines 345 and 418 in our submission containing tracked changes) and reworded our findings to be more direct.

**Page 12, line 271 –In Figure 1B, it seems to me that the 7-day virgin NBF has only three points. It is not indicated how many replicates were done for this data set in particular. If it were the same number as for panel 1A, was there one experiment where there were no survivors at this time? If this is the case, this would not be inferred from Figure 1A.**

Response: Sample sizes were similar for all treatment groups in this experiment and have been added to the figure legend. The reason there are only three points for 7-day virgin NBF in fig. 2B is not because there were only 3 individuals used in the experiment, it is because fig. 2B shows count data (i.e. individuals with >0 CFU) and most 7-day virgin NBF females had zero CFU (as can be seen in fig. 2C). We have edited the text in the figure legends to clarify these points. Additionally, we have included raw data in supplementary file 5 for full transparency.

**More importantly, I believe that the bacterial load data of the blood-fed females could have been an extremely important data to have. Considering the exponential growth of bacteria in the mosquito gut during the blood digestion and the immune modulations known to occur during this time, attributing the deaths to the experimental infection or not can’t be done cleanly without knowing the bacteria load in these individuals. Maybe the Serratia growth didn’t burst PBM but facilitated the leakage of other bacteria from other bacteria.**

Response: We agree that bacterial load data would be extremely beneficial for this dataset and have collected it in Figure 4. We specifically chose to examine bacterial load at 16 hours because of the high drop in survival seed in all experimental groups after 24 hours (figure 2). We attempted to collect bacterial load data for BF individuals for Figure 2, but given the extremely high mortality rates in blood fed individuals at this dose, we were unable to collect these data. Our figure 4 (low dose) results show that blood fed individuals have significantly decreased survival post infection (p = 0.003), but do not have significantly higher bacteria loads (p = 0.525) as compared to non-blood fed females. This suggests that other factors are at play which we did not examine, such as decreased BF group tolerance, or microbe-microbe interactions associated with microbes introduced from a blood meal. While we did not examine bacterial load at different digestion time points post-blood meal, we agree that this is valuable to consider and should be the subject of future studies.

**Page 13, line 284 – with the degree of variation described in the pricking method used for this experiment, the difference between the two infecting doses does not look significant enough to be able to identify dose-dependent effects.**

Response: Please refer to our previous response addressing Page 9, line 191. We have thoroughly re-evaluated and clarified our dosing designations with greatly improved accuracy. We appreciate the keen eye of this reviewer, as it allowed us to correct a regrettable mistake.

**Page 17, line 343 – The format of the legend for the different panels is not consistent with the one used for the other figures.**

Response: This has been edited.

**Reviewer #2: (No Response)**

**Reviewer #3: (No Response)**

**________________________________________**

**Summary and General Comments**

**Use this section to provide overall comments, discuss strengths/weaknesses of the study, novelty, significance, general execution and scholarship. You may also include additional comments for the author, including concerns about dual publication, research ethics, or publication ethics. If requesting major revision, please articulate the new experiments that are needed.**

**Reviewer #1:**

**I would like to commend the authors for the work done and I do believe it is a very relevant field of study, with many questions still needing further exploration. I have explained my reservations about the methodology and I hope they are seen as they were intended: constructive criticism. The writing in the paper is in general very clean and well redacted, but I would suggest a deeper search into the literature up to date, to really put together what are the gaps of knowledge regarding the links between mating and immunity in Aedes aegypti.**

**To claim that mating affects "immune responses", immune responses must be studied, and not only survival or infection prevalence. While I see the benefits of working with whole organisms, it is important to understand that signals triggered by mating affect different tissues in different ways and at different times. Within a same tissue, different cell types could potentially be differentially modulated by different signals coming from the mating event. To unequivocally say that mating impacts immune responses, and in a different way as previously described in many previous of studies, experiments including different components of the immune responses must be made, and the experimental groups must be prepared to guarantee beyond the shadow of the doubt that the virgin females are really it.**

Response: We greatly appreciate the time and effort you have put into providing constructive feedback. We have attempted to address your concerns and believe the changes have made the manuscript much stronger.

**Reviewer #2:**

**This study investigates the effects of mating and blood feeding on the immune defense of female Aedes aegypti mosquitoes. The authors found that mating increases survival and bacterial load under medium to high doses of bacterial infection, whereas blood feeding consistently reduces survival across all infection doses.**

Response: We greatly appreciate the time and effort you have put into providing constructive feedback. We have attempted to address your concerns and believe the changes have made the manuscript much stronger.

**Reviewer #3:**

**Previous studies in other insect systems have shown that mating can impact the hosts response to infection. In some systems this effect is beneficial while in others detrimental. This has not been empirically tested in Aedes aegypti which is significant because blood feeding is intimately linked to reproduction and blood feeding has been shown to alter mosquito responses. The authors tested whether mating and/ or blood feeding had a significant effect on infection outcomes after exposure to different doses of a pathogenic bacterium, Serratia marcescens. Overall, the experimental design was a little convoluted and could have been better explained, possibly through the addition of a experimental design figure. The analysis of the results seems appropriate but the presentation of the data in the figures could be improved, specifically adding significance demarcations and sample sizes. The finding that mating increased survivorship was significant and adds to the literature; however, the title and discussion are focused on immunity yet there is no data supporting the claims that immunity is important in this phenotype. Further, the authors present conflicting data, i.e. increased survivorship in mated individuals but also significantly higher bacterial counts, which argues against an immune-based mechanism. These contradictions are not discussed. Lastly, in order to draw links to the immune response additional data is needed, the very least being gene expression profiles of antimicrobial genes.**

Response: We greatly appreciate the time and effort you have put into providing constructive feedback. We added an experimental design figure and have added additional detail to the figures and legends to address your concerns. We have also added discussion of the contradiction you point out – which we think is likely indicative of a tolerance effect – on lines 404-410.
